# Alabama Dental Association

**Published:** 1894-09

**Authors:** J. M. Walker


### THE DENTAL REGISTER.

VOL. XLVIII.] SEPTEMBER, 1894. [No. 9.

### PROCEEDINGS.

### Alabama Dental Association.

Reported for the DENTAL REGISTER by Mrs. J. M. Walker.

The twenty-fifth annual meeting of the Alabama Dental Association was held in Montgomery, April 10 to 13, 1894.

OFFICERS: Dr. T. M. Allen, Birmingham, President; Dr. R. A. Rush, Selma, First Vice-President; Dr. W. E. Proctor, Sheffield, Second Vice-President; Dr. G. M. Rousseau, Montgomery, Treasurer; Dr. S. W. Foster, Decatur, Secretary.

After prayer by the Rev. Mr. Davis a cordial welcome was tendered the members of the association, to which graceful response was made by Dr. Chas. A. Merrill.

The president then delivered his annual address. He addressed himself especially to the younger members of the association, urging upon them the importance of continuous labor and study if they would achieve the professional rewards of honor and distinction. As appropriate to the quarter-centennial of the association the president embodied much statistical and historical matter in his address. He pointed out the fact that of the founders of the association but four survive. Two of these, Dr. H. D. Boyd and Dr. H. Locke, are still active members. Dr. Allen also reviewed the history of dental legislation in Alabama, and the work of the State Board of Dental Examiners. He urged upon the association the appointment of a committee to secure such legislation as would prevent the indiscriminate extraction of teeth by street fakirs, Indian doctors, etc. This portion of the address was discussed at some length by the association. Dr. Allen called attention to the injurious effects of the nostrums used by these irresponsible individuals to secure so-called "painless extraction" resulting in swelled faces, sloughing, necrosis and other serious injuries. Within three weeks after a visitation of one of these peripatetic tooth-pullers he had had seven cases of necrosis to attend to. He said, this is not a matter of dollars and cents, but of irreparable injury to the people who, in their ignorance, patronize these charlatans.

Dr. W. H. Morgan, Nashville, Tenn., said this state of affairs was a disgrace to the dental profession; this class of men brings dishonor upon an honorable profession. Their brass bands and gilded chariots and especially their low prices are irresistible to the lower classes by whom they are classed as veritable *dentists*, and the injury they do is reflected back upon the dental profession. It is an outrageous practice—an inhuman butchery—which should not be tolerated by law. He had seen several cases in which the local injections had caused induration of the tissues, a slow, cold, inflammatory action of which the final result cannot be prognosticated. Many well-meaning dentists, anxious to relieve their patients from the pain attendant upon tooth-extraction, buy and use these patented nostrums of which the formula is kept secret, using them without evil intentions, but, nevertheless, in violation of the code of ethics. Every provision of the code is justified and should be obeyed. It is the duty of the dental profession to look after this thing and have it prohibited.

The president in his address also urged that a representative from the State Board of Dental Examiners be sent to the meeting of the National Board.

Dr. J. Y. Crawford, Nashville, Tenn., hoped that this would be done. He said that the two National Associations of College Faculties and Examiners, working together in harmony, had done very much toward elevating the dental profession. He said that it is very desirable to bring about harmony and unity of action between the different State Boards and to secure unification of the State Dental Laws, or trouble would arise from efforts to have those laws repealed. Before laws are enacted or amendments asked for, the questions should be asked—is it just? is it wise? does it seek to protect the few, or is it for the benefit of the whole people? All such laws should be placed on the statute books in terms of the plainest statement of fact. In trying to draw up laws to meet every possible emergency it gets to be so complicated that neither the people nor the officials can determine the meaning. Our own State law has no provision interfering with this promiscuous extraction of teeth, because we feel that in making a start we could not ask for everything; but it is not right. It brings about a condition of things necessitating eventually an expenditure of hundreds of dollars in remedying the evils done in saving on the cost of legitimate tooth-extraction. The laws must be so amended as to punish this indiscriminate extraction by irresponsible persons. (A voice, "You won't get the amendment.") In saying we won't get it, you do more to hinder it than anything else. If we get the unanimous consent of the State Societies and of the State Boards—if every dentist says *it must be*, if no one pulls back but all push forward, in less than five years we will have it on the statute books. Talk to your intelligent legislator and show him that he would be derelict to his duty if he fails to support such a measure. Any man who cannot be made to see his moral duty in such a case had better be retired to private life. In union there is strength, and I have sufficient confidence in the love of right and the moral integrity of our legislators to say *it will be done.*

At the close of the discussion a committee was appointed to secure the enactment of a law prohibiting the extraction of teeth "for pay or reward" except by those who have been before the Board of Examiners and obtained license.

On motion of Dr. S. W. Foster, Dr. T. P. Hinman, Atlanta, Ga., was elected an honorary member of the association, and was invited to describe his method of securing a perfect adaptation of the Logan crown to the abutment of the natural root, as follows: The root having been properly prepared and trimmed, a small piece of paraffine is molded around the pin, close up to the porcelain. A small disk of No. 60 tin-foil or platinum is then cut, a little longer than the abutment of the root, and threaded on the pin close up in the wax. The crown is then placed on the root, the pin having been bent, if necessary, to secure alignment, and forced closely into place, the wax driving the foil disk into perfect apposition with the abutment of the root, of which it receives a perfect imprint. Removing carefully the crown, the wax and foil in position, with a warm blade, all the excess of wax beyond the circumference of the root is trimmed away and the projecting margins of the disk snipped with sharp, fine-pointed scissors, at intervals to the imprint of the abutment. A small pellet of wax is then placed on the point of the pin, and the whole invested, point down, in quick-setting plaster just up to the porcelain. When the plaster has set, remove the crown, the snipped edges of the disk securing the latter in the plaster, leaving a perfect metallic-surface model of the abutment of the root, exposing to view every surface in all its diameters. The crown being cleaned of the wax is then ground to the root expeditiously and accurately, the pellet of wax which was placed on the end of the root allowing space for the pin to penetrate deeper in the plaster model of the root, as the porcelain is ground away. This method will be found applicable to all of the teeth for which Logan crowns can be used, and for all porcelain crowns which have platina pins baked in them.

Dr. Merril expressed the thanks of the association for this eminently practical method of overcoming what sometimes proves to be a great obstacle in the use of the Logan crown, and gives an added value to this invaluable crown.

Dr. T. M. Allen said that he had tried this method and found it practical and valuable.

Dr. J. Y. Crawford said that this one item was sufficient to repay him for his time and trouble in coming from Nashville to Montgomery.

Dr. J. D. Adair described the method of using the kalium natrium (potassium-sodium) preparation of Dr. Emil Schrier, and reported very satisfactory results in a number of cases. In one case he had vainly treated for six or eight weeks abscesses of three superior incisors, which were to receive Logan crowns. As the young lady had to return to her home in Baltimore, he concluded to make an application of the preparation and fill the roots without further delay. There was still pericemental tenderness with pus constantly present. The Logan crowns were put on immediately after one treatment with Dr. Schrier's preparation, and the young lady left town the next day. He awaited the result with considerable apprehension but was gratified to learn that the treatment was perfectly satisfactory. Ten months have elapsed since the crowns were put on, and the roots have given no trouble.

Methods of obtunding sensitive dentine were discussed at some length, especially of teeth that are to be shaped for crowning where the operation cannot be completed at the first sitting.

Dr. T. M. Allen finds ammonia and hot burnishers of great assistance.

Dr. Chas. A. Merril uses the old stand-by, chloride of zinc. He uses it when merely deliqusced, getting the escharotic action constringing the contents of the tubuli. When a layer of dentine is removed another application may be made, and so on until all the desired cutting is effected. It may give some pain, but less than by other methods. A gutta-percha cap molded over the tooth will keep it comfortable until the next sitting.

Dr. W. G. Browne, Atlanta, Ga., uses opiates, getting the patient into such a condition that he is both willing and able to bear the necessary pain.

Dr. S. W. Foster places in contact with the dentine a pledget of moistened cotton on which he has taken up some crystals of nitrate of silver, holding it in contact with the tooth by means of a gutta-percha cap molded over the tooth.

Dr. C. L. Boyd uses nitrate of silver.

Dr. H. D. Boyd finds the value of nitrate of silver greatly enhanced by moistening it with alcohol instead of water.

Dr. Watson, finding every other application fail, applied arsenic to the sensitive dentine, expecting to have to remove a dead pulp later on. The patient did not return for more than a year and then reported everything satisfactory. Feeling satisfied that the nerve must have died from the arsenical application he removed the filling but found the dentine so sensitive that the patient could not allow of any excavating. He therefore refilled the cavity, and after eight years' service it is still all right. If it is dead it shows no signs of death.

DR. W. G. BROWNE described an interesting case in orthodontia; interesting because of the conditions, the method of treatment and the results. In this case the upper front teeth projected so far that they closed over the lower lip, the teeth being very long and very thin; the lower front teeth struck the soft tissues at the palatine surface of the upper incisors. The chin was very receding, forming almost a straight line from the mouth to the neck. After careful study of the case he decided that the deformity was due—not to protrusion of the upper jaw but to lack of development in the latter. He placed in the mouth a plate covering the roof, so formed as to allow the lower incisors to strike on an inclined plane, and to hold the very short molars apart one-fourth of an inch. The day the plate was put in the family went to New York and he did not see the patient again for six months. When he next saw her he first had a view of her side face and could scarcely believe it was the same child, so great was the improvement. The chin had elongated till the face was perfectly symmetrical. On examination of the mouth he found the "bite jumped" one whole tooth; the molars had elongated till they were in contact and the lower incisors passed up only one-half the length of upper incisors—all of the teeth and the mouth being now in normal condition; all effected in six months, without any attention, simply by wearing the plate described. It had made a wonderful and radical change. The patient is now fifteen years old and a very pretty girl. In reply to a question, Dr. Browne stated that he did not obtain the history of the deciduous teeth or whether they had been prematurely extracted. Several members of the family had abnormal dental developments—varying peculiarities. An uncle had an exceedingly projecting under jaw with very short molars, the inferior incisors closing up outside of the upper teeth.

DR. C. L. BOYD said it was the imperative duty of every dentist to instruct patients in regard to the importance of the sixth-year molars, not allowing them to be lost under the mistaken idea that they will be replaced ; they should be taught how much depends upon them for comfort, regularity of the teeth and symmetry of the face.

Dr. Orr showed a rubber plate made very thin by the "Morris" patented method and strengthened by a band of gold vulcanized in the plate. To this band were soldered (before vulcanizing) bits of gold plate between the teeth, which being trimmed to proper shape and burnished to the tooth gave the appearance of the gold fillings which patients sometimes desire in artificial teeth—a much easier method than drilling cavities in porcelain teeth.

Dr. Chas. A. Merrill obtains the same effect by cutting a little channel in the mesial or distil aspect of the tooth with a diamond disk, which he fills with plaster. After vulcanizing he removes the plaster, drills a retaining pit in the vulcanite and fills the cavities in the teeth. This is very easily done, as there is no risk of moisture or *sensitive dentine.*

Dr. J. A. Frazier explained his method of "taking the bite" where the patient protrudes the chin, as is so often the case. After the wax is in the mouth, he places one hand on the back of the head and the other over the chin, exerting a strong pressure at the moment the mouth is ordered closed.

Dr. J. E. Frazier, in difficult cases, stands behind the patient and places the thumbs in the sigmoid notch.

Dr. W. H. Morgan, Nashville, Tenn., said there was no method of taking the impression for an articulating model that is of universal application. The ligaments which control the condyle are very lax in some cases, and strongly elastic in others. Some can draw the jaw much farther back into the glenoid cavity than others. With some patients it is almost impossible to get them to relax the masseter muscles.

A very good method with some is to direct the patient to throw the head back and swallow at the moment of biting into the wax. With some it is impossible, with all your efforts, to get anything but a "jimber-jawed" bite, and after the teeth are waxed up, you have to rearrange them to fit the mouth.

Dr. Merrill spoke of the importance of correct nomenclature in discussing prosthetics. He said: We talk about impressions and molds and models and patterns and casts in a very confused and confusing manner. The proper nomenclature of the process of constructing an artificial denture is first the impression taken from the mouth; then the cast obtained by pouring plaster into the impression; then the model built in wax on the cast; then the mold left after removing the wax, into which is pressed or poured the material of which the plate is constructed.

Dr. A. A. Pearson related an amusing case showing the importance of being careful in the terms we use with our patients. He was making plates for an old lady who was very difficult to please. She objected to having the teeth tried in on the wax base plate, which he unfortunately broke in removing from her mouth, necessitating a second impression. He could only persuade her to have another one taken by assuring her that the first one was only "*temporary*" and that he must take a "*permanent impression.*" Misfortunes never come singly, however, and after completing the plate he dropped it, stepped on it, and all his labor was lost. The old lady refused to have another impression taken, and left in very ill humor to go somewhere else.

The second dentist considered himself very fortunate, with such a patient, in getting everything correct from the first impression, but when he put in the finished plate, she absolutely refused to accept a plate made from "*a temporary impression,*" saying she knew more about dentistry than to allow that sort of work to be done for her!

So she went back to the first dentist who, though he might want a third impression, did not attempt to palm off a temporary impression on her!

Dr. O. C. Farish read a paper entitled "Physiological Chemistry and Therapeutics." In this paper he reviewed briefly some of the universal phenomena of physiological chemistry, as the absorption of new material and the throwing off of effete matter; the incessant removal of organized material; the direct relation between the quantity of material consumed and the active manifestations of life; the material conditions necessary to life, as the consumption of oxygen and the discharge of carbonic acid; the presence and absorption of moisture; the definite limits of temperature; the specific proportions of certain chemical ingredients and the parts taken by them in the act of nutrition, in giving special character to the different organic tissues and fluids of the body; the way in which they are supplied and the changes which they undergo within the body; the phenomena of endosmosis and exosmosis, and the function of chloride of sodium in this wonderful process, and many other interesting points.

Passing on to special therapeutics, the writer dwelt especially on the importance of noting the pathological conditions, not only of the mouth, but of the system generally, and the necessity in many cases of systemic treatment before local treatment can become available or of any benefit. In treating pathological conditions he urged the prime importance of first ascertaining and then of removing the causes of irritation.

This last point was made the special feature of the discussion which followed the reading of the paper.

Dr. W. H. Morgan said all cures depended on the removal of the cause or causes of disease—first ascertain, then remove the cause. In oral diseases, however, it is difficult to ascertain the cause; especially is the cause of caries obscure, and even when we can detect it we cannot remove it. We do not treat abscesses; we simply remove the cause—nature does the rest.

Dr. J. Y. Crawford, Nashville, Tenn., (honorary member) dwelt at some length on the importance of treating from a constitutional and systemic standpoint. He said: We are not doing our whole duty when we assume to treat and manage as a specific affection that local form of disease known as dental caries; it is a pathological factor of systemic origin and from local surroundings and environment. Away, back of, and antedating present conditions, are etiological factors found in the organism. There is hereditary transmission of caries from father to child—a physical dyscrasia, susceptibility, is present in marked degree. What is the most common cause of secondary or recurrent decay around fillings? There are etiological factors that cause failures of fillings from the hands even of master operators. There are conditions of pericementitis due to physical dyscrasia that you may treat *locally* for days and weeks in vain, till you resort to constitutional therapeutic treatment. In pulpitis the nervous system is involved; constitutional conditions aggravate it. You must control the heart's action; anodynes alone are of no service; the pulpitis remains unchanged. But give a heart-sedative, lessen the power of its action, and then your anodynes will prove of avail. Therefore, I say, follow out the suggestions of the paper; look after the preceding pathological conditions that led up to the local trouble. We have learned to look for causes back in the organism, back of the tissues involved in the metabolism. We restore equilibrium and build up; through the lines of chemical therapeutics and physiology much may be accomplished. If the question of the etiology of diseases pertaining to the mouth is solved by odontological science, it will hold a position paramount to all other sciences.

Dr. L. D. Carpenter and Dr. Wm. Crenshaw, both of Atlanta, Ga., addressed their remarks to the younger members of the profession, emphasizing the thought that the great work of the future lies in their hands with the increased advantages offered students in these latter days. So many duties crowd and press on the older members that they are not able to give the time and the careful thought these investigations demand.

Much in pathology that is puzzling and vexing now will be made clear, and easily handled, as the result of the work and the investigation of the younger men now taking up this great work.

Dr. J. D. Adair read a paper entitled "Ignorance and Negligence of Parents in the Care of Children's Teeth." This paper was an endeavor to formulate some method of reaching the public—other than by personal instruction at the chair—which shall not be open to the charge of violation of the code of ethics, as a form of self-advertising. The plan is a pamphlet, to be gotten up by a committee appointed by the association, and issued by the association for general distribution.

Dr. J. T. Stuart endorsed the paper, and emphasized the need of something more than the laborious and unsatisfactory method of individual effort. He said: "We have not time to give a course of hygienic lectures to every patient."

Dr. C. L. Boyd endorsed the plan suggested, and thought that endorsed by the association it would have a powerful effect on the public, and also tend to elevate the profession in the eyes of the public, showing them that we are not working solely for the dollars, nor aiming only at repairs of damages, but that we are able to offer means of prevention as well as of relief. He thought it would be well for the pamphlet to include a formula for a safe and effective dentrifice for those who do not go to the dentist, but who go to the drug store, and buy, in their ignorance, that which is worthless, if not positively injurious.

Dr. W. G. Browne thought such a pamphlet would reach only those whom we reach in our offices; that the general press is the best medium through which to reach the general public, short, terse, well-written articles on subjects calculated to interest the public will always be accepted by your daily or weekly newspapers, and will be read by those who patronize quacks, because they read their advertisements and believe in them. If you want to reach your patients, give them Mrs. Walker's book, "Letters to a Mother," that will teach them all they need to know about their children's teeth, but if you want to reach the general public you must do it through the public press.

Dr. Morgan spoke in commendation of the pamphlets in this line, one issued many years ago by J. W. White, and another more recently by "Mrs. J. M. Walker," well adapted to the purpose.

Dr. Williamson thought every dentist ought to have just such literature ready for presentation in reply to the questions that are asked, and which the dentist has not time to answer in detail while busy at the chair—questions which are suggested to the mother or the friend by the work in hand. We must educate the parents for the sake of the children.

Dr. W. H. Morgan thought it a waste of labor to endeavor to educate people who are not seeking education; that it is obtrusion to force knowledge on those who do not ask for it; if they do not desire information they will not appreciate it, while those who are already well-informed will read only to criticise. One case of severe toothache which you relieve will do more good than all your teaching in convincing the mother that dentistry has resources to cure and to prevent in the future. If the dentist secures, by his personal conduct, the respect and confidence of the public they will go to him for the care of their teeth, but no system of literature will do this.

Dr. A. S. Billings, Omaha, spoke of the value of literature of this class—not for distribution among patients but for the friends of patients, to occupy their time while waiting. Put something of this kind where they can see it, and get them interested in the subject of the teeth.

Dr. J. Y. Crawford thought it would be perfectly useless to try to teach the public the principles of any profession whether law, theology, medicine or dentistry; teach them that the professors are reliable men; that they know their business—that is all the public need to know. Therefore, such efforts as the paper proposes, whether through the public press or through pamphlets, will never accomplish much. And so we stagger along trying to reach the solution of the vexed question. Dentistry has been in existence, in a broken line, for over three hundred years, and yet the combined influence of all those years has not been strong enough to teach mothers that the first permanent molar comes in at the age of six years, and that when lost it will not be replaced by another. We are all agreed on that fact, and we make dogmatic announcement of it, and that it should not be lost by decay or removed. We teach mothers that they must not allow it to be extracted as it is the most important tooth in the jaw. But if a child is brought to me suffering from toothache and I want to save it, if the mother declines my offer, how far will she have to go to find some *dentist* ready to take it out? Dr. Morgan told the truth when he said that the logic of one successful operation was more convincing than all the arguments in literature. The logic of facts is irresistible and one filling that lasts for years speaks more than volumes, and in a language that the patient can understand. Every child born into the world ought to have the services of a dentist from the eruption of the first tooth throughout the entire life. Then we would have proper opportunities for observation. Dr. Crawford spoke at some length on the question of the relation between infantile mortality and dentition which he pronounced a most towering question—a most vital question, and one in which the tender heart of the mother is most interested. He said: Dentition is a physiological function, but alas! it is not performed physiologically in this age and country. I am one of those who believe that any departure from normal lines in tooth-evolution and tooth-eruption is largely responsible in a large percentage of infantile mortality. I ask you all to unite with me in this one thing—say to the American people: No child should be allowed to live with dental decay in the mouth; take this disease out of the mouth. No parent is excusable in permitting it to remain.

The subject of ethics was discussed at some length.

DR. CRAWFORD said: When a young man receives his diploma from a reputable college he voluntarily assumes the obligation to do *what is right* in all his professional relations. In entering into these relations he proposes to confer benefits on humanity and unless he lives up to this he violates his highest moral obligations.

DR. H. D. BOYD gave, as the simplest definition of ethics, the golden rule, "Whatsoever ye would that men should do unto you, even so do ye unto them." There is no broader, deeper, truer definition of ethics than this.

DR. THOS. P. HINMAN, Atlanta, Ga., read a paper on "Aluminum" giving a brief history of the metal and its uses, especially in dentistry. He described the method of using aluminum in making crowns by the "Morrison seamless method," thickening the cusps with amalgam worked very dry. In vulcanizing rubber plates Dr. Hinman substitutes aluminum for tin foil for giving a polished, finished surface, as it gives no discoloration as when tin foil is used. Also as a matrix for amalgam and cement fillings, oiling the surface slightly; it is so soft that it can be formed around the tooth very perfectly. At the conclusion of the paper

Dr. Hinman passed around for inspection some shell-crowns soldered as for bridge-work, and also some small pieces of plate-aluminum united at the straight edges, with the aluminum-solder furnished by the Wilmington Dental Manufacturing Company. He stated that he had not tested the solder in the mouth and could not say how well it would resist the action of the buccal fluids.

Dr. W. G. Browne, Atlanta, Ga., spoke very favorably of the aluminum crowns. In striking them up he places wax with cotton over it in the blank for stamping the cusps and finds that it gives a fine, bulging contour.

Dr. A. S. Billings spoke of the mistake commonly made by dentists in using aluminum plate too thin and also in not taking sufficient pains to prevent *leading*. Aluminum for plate-work should not be of less than twenty guage; if gold-plated it will not disintegrate.

Dr. T. M. Allen said that thin tissue paper placed on each side of the aluminum in swaging will effectually prevent *leading* and obviate the necessity of oiling the metal which blackens it. A pickle of chemically-pure sulphuric acid bleaches it perfectly, with a frosted appearance which is very attractive. He uses the Fenner furnace and the metal furnished in ingots with the furnace which makes a very succesful casting.

Dr. Crawford prefers a low carat of gold for cheap crowns, as the necessary thickness of aluminum makes them cumbersome. For lower plates Dr. Crawford thinks there is nothing equal to aluminum swaged between Babbitt's metal dies. The plate must be carefully articulated, and not less than sixteen or eighteen Stubbs' gauge. Dr. Crawford described his unique method of swaging aluminum, as follows: Take an accurate impression and make a sand-mold and metal die. Then cover the plaster model very accurately with sheet paraffine-wax, and from this wax-covered model make another sand mold, metal die and counter die, the counter die being thus enlarged by the thickness of wax with which the plaster model was coated. Swage the aluminum between the first metal die and the enlarged counter die, and the resulting plate will be very satisfactory.

Aluminum is very kind to the mucous membrane and will not cause recession and absorption of the alveolus.

Dr. J. T. J. Watson read a paper entitled, "Dentistry—Past, Present and Future," which gave rise to a lengthy discussion of the comparative merits of cohesive and non-cohesive gold and the merits and demerits of amalgam.

Dr. C. L. Boyd read a paper on the "Etiology of the Irregularity and Crowding of the Teeth," attributing this largely to two factors—first, undeveloped maxilla, due to lack of exercise of the parts, and second, premature extraction of the deciduous teeth. To remedy the first, Dr. Boyd outlined a system of what might be termed mouth massage, a systematic but gentle rubbing of the inside of the infantile jaws by the mother or nurse, by which he claims the jaws may be so developed as to allow the deciduous teeth to erupt with sufficient proximal space to prevent decay, allowing them to be shed normally, with ample room for the permanent teeth.

In addition to the benefit derived from this passive exercise, the child is thus accustomed to having the mouth handled, and when the time comes will not object to having the teeth washed at first, and then brushed, and will submit willingly to the proper examination and care of the teeth.

For the second he would have the public, and especially mothers, educated in the proper care of the teeth.

Dr. J. H. Crossland read a paper entitled "An Original Crown."

For the crown descibed by Dr. Crossland, the root is prepared as usual for an ordinary porcelain crown. With a very fine drill, preferably one with a gauge, a series of holes is drilled as near as practicable to the circumference of the root and the interspaces cut out, forming a groove in the face of the abutment. A band of 28 or 30 gauge, 24-carat gold, is fitted into this groove and ground off to the base of the root. To the band is soldered a flat cap of pure gold, 34 gauge, using as little solder as possible, 20-carat. This is replaced in position and burnished to the face of the root. It is then punched and a platinum post placed in position, secured with hard wax, inverted and soldered, the cap being trimmed accurately to the outlines of the root end. A plate tooth is then backed with gold as follows: The tooth is ground at the gingival end to fit the cap and beveled from pins to cutting edge. From 34 gauge, 24-carat gold, a backing is cut slightly longer than the tooth which is punched, placed in position in the tooth, and held by bending the pins. The gold is burnished thoroughly to the tooth and slightly over the square end or bevel of cutting edge.

Another backing is cut, long enough to burnish over the articulating edge to thicken the gold at this point, in which a number of holes are punched to insure perfect union in soldering.

All the parts being placed in position, it is carefully waxed up and invested, as usual, for soldering.

Dr. Crossland claims for this crown that it is less bulky and more shapely than the old ferule crown; exposes no gold at the gum margin; allows a much more favorable and accurate adjustment with regard to other teeth; can be used when convergent or divergent roots are to serve as abutments for bridges; is especially adapted to conically-shaped roots; causes less pain in adjustment and will not irritate the pericemental membrane. It is very cleanly, as the joint between gold and tooth can be made about perfect. The band and cap may be made of platinum and a porcelain backing baked, producing a highly æsthetic crown.

Dr. R. A. Rush read a paper entitled "Dentistry as a Specialty of Medicine," the special points of the paper being the question, "Does the practitioner of medicine realize and recognize the great work we are doing in the prevention of disease, and the still greater work we could do with his cordial co-operation?" his conclusion being—"I don't believe he does, for if he did he would not howl so about the sanitary regulations of a city, and yet try to treat patients successfully with their mouths full of carious teeth, alveolar abscesses and other pathological conditions which render it a habitat for those germs which science has proven are deleterious to health."

In the discussion of this paper Dr. Crawford said: Magnify the idea, strengthen it, that dental surgery is a department of general medicine and surgery. In our discussions of this subject we hear much that seems to be a criticism of the intelligence of physicians. But who is responsible for the inability of the physician to diagnose the diseases of the oral cavity? I say *we* are responsible for their ignorance. It is only the legitimate sequel of our own failure in duty, and it reverts back on us to our disgrace and shame. Of all the needs in our institutions of learning the greatest need is a full chair of dental and oral surgery and public hygiene. Think for a moment and compare the value of dental surgery and ophthalmic surgery! Dentistry is the only department that touches the human system from one end of the line to the other. There is no other branch *save one* that touches the head, the heart and the life; we must all *be born*, and we all *have teeth*, but we *don't* all go blind or deaf! I hope yet to see in every medical school one member of the faculty whose duty it shall be to teach what the people need in dental surgery, and what dental surgery comprehends.

Dr. W. H. Morgan thought the charge made by Dr. Crawford not justified by facts. He said: We have not "hid our light under a bushel." We have been prolific in literature, we have text-books in the language of every civilized nation; these are at the command of medical men. We are open and free and frank and ready to impart our knowledge. Dentistry does belong to the healing art—but of medicine? No! They shut out; they refuse to consult with us; they elbow and crowd and try to keep us out.

Dr. Rush replied that he had been invited to read this paper before his own County Medical Association.

Dr. Crawford maintained that his position was correct—that while medical men are instructed in the principles of ophthalmology yet they are not familiar with the etiological factors of alveolar abscess, which is one of the most common conditions they have to diagnose; they cannot differentiate between pericementitis and pulpitis. He said, every D.D.S. is entitled to membership in any medical association, and you want to have membership in medical societies. You would then be enabled to read papers on the pathology and etiology of oral diseases; then they would look into the surgical principles involved and you could get them to see *there is something* in dental science. We want to establish our relationship with them, not for the sake of advancing theories but for the good of the people. I hope you will join your local medical societies; you can give information and you can receive help.

DR. MORGAN: I can say *amen* to all that.

DR. E. M. ROUSSEAU: The constitution of the Alabama Medical Association does not admit to membership any but those holding the degree of M.D., but all dentists are invited to attend meetings, and can be elected associate members, read papers and take part in discussions. In many cases we are called in consultation, but it is not in every community that physicians are so cordial.

This concluded the papers and discussions of the meeting.

The election of officers resulted as follows: Dr. H. D. Boyd, Troy, President; Dr. O. C. Farish, Camden, First Vice-President; Dr. H. B. Williamson, Evergreen, Second Vice-President; Dr. S. W. Foster, Decatur, re-elected Secretary; Dr. G. M. Rousseau, Montgomery, re-elected Treasurer; Drs. E. Wagner, Montgomery; P. R. Tunstall, Mobile; J. S. Bailey, Demopolis, Executive Committee.

The association adjourned to meet in Mobile the second Tuesday in April, 1895.

